# Optical properties and ionic conductivity studies of an 8 MeV electron beam irradiated poly(vinylidene fluoride-*co*-hexafluoropropylene)/LiClO_4_ electrolyte film for opto-electronic applications

**DOI:** 10.1039/c8ra00970h

**Published:** 2018-04-23

**Authors:** Yesappa L., Niranjana M., Ashokkumar S. P., Vijeth H., Basappa M., Jishnu Dwivedi, V. C. Petwal, Ganesh S., Devendrappa H.

**Affiliations:** Department of Physics, Mangalore University Mangalagangothri-574199 India dehu2010@gmail.com; Head, Industrial Accelerators Division RRCAT Indore-452013 India

## Abstract

The influence of 8 MeV energy electron beam (EB) irradiation on optical properties and ionic conductivity of PVDF–HFP/LiClO_4_ (90 : 10 PHL10) electrolyte film with 40, 80 and 120 kGy doses. The FT-IR results show that C=O bond stretching at 1654 cm^−1^ is due to the degradation of polymer chains and the CH_2_ bond wagging intensity at 1405 cm^−1^ corresponds to C–H bond scissioning in the 120 kGy dose irradiated film. ^1^H and ^13^C NMR spectroscopy was performed and the ^13^C NMR spectra confirm the effect of EB irradiation of the PHL10 polymer electrolyte by sharpening and splitting the spectral lines with increasing EB dose and revealing a new spectral line at 162.80 ppm with a 120 kGy EB dose. The size and shape of the porous morphology was drastically changed, becoming deeply porous with a visible inner hollow shaped structure, suggesting increased amorphous character upon irradiation. The absorption band of the unirradiated film observed at 202 nm in the ultraviolet region is shifted to 274 nm after irradiation due to inter band transition of electrons from the valence band to the conduction band and the optical band gap decreasing from 3.49 eV in the unirradiated film to 2.64 eV with a 120 kGy EB dose. Segmental motion in the polymer matrix leads to a decrease in the local viscosity by increasing the mobility of ions upon irradiation. Nyquist plots show semicircles at high frequency due to Li-ion migration through the porous surface of the electrolyte film. A maximum ionic conductivity of 8.28 × 10^−4^ S cm^−1^ was obtained with a 120 kGy EB dose and the observed cyclic voltammetry of the irradiated polymer electrolyte suggests it is electrochemically stable.

## Abstract 2

A study into the effect of 8 MeV energy electron beam irradiation on the optical properties and ionic conductivity of a PVDF–HFP/LiClO_4_ electrolyte film.

## Graphical abstract

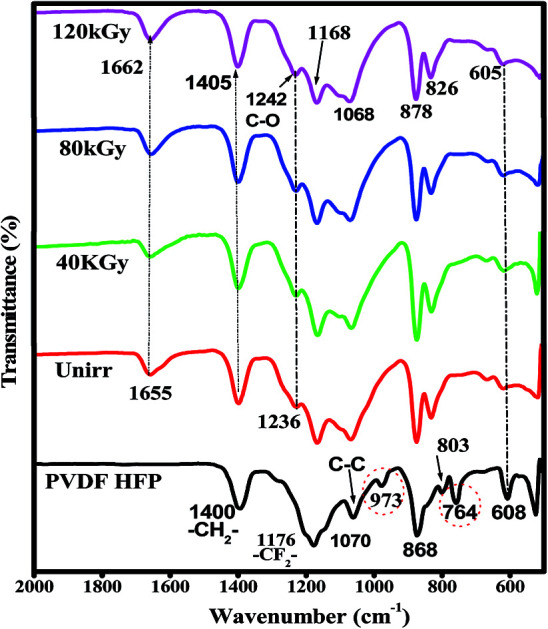

## 1. Introduction

Irradiation techniques are rapidly developing as advanced methods for modifying materials’ physical properties. There are various types of radiation such as ion, electron, gamma, neutron, ultraviolet, X-ray *etc.*^1^ Among these, electron beam irradiation is optimised and produced by an accelerator; whenever it interacts with a polymer, the polymer undergoes radiation processing with the result being a breaking of chemical bonds or polymer degradation occurring in terms of cross linking and chain scission.^2^ The process of cross-linking in polymers usually results in the joining of adjacent polymer chains to form a three dimensional network in which radicals recombine with each other. Chain scission occurs whenever the radicals fail to recombine, reducing the molecular weight. Cross-linking and chain scissioning depend on the chemistry, structure, and morphology of the materials and chemical alterations that may reorient the chemical bonds directly influence the physical properties of the polymers.^3,4^ Therefore, treatment with EB radiation is a promising approach to control the modification of polymers’ physical properties in order to use them in various applications. EB radiation has been efficiently applied in industry to promote cross-linking and chain scission in polymeric materials for use in various applications like insulation on wires, packaging, parcels *etc.* Hence, it is necessary to understand the influence of EB radiation on physical properties of polymer electrolytes to enhance the electrical conductivity and optical properties for exploiting their many potential applications such as solid-state batteries, opto-electronic devices and electrochemical applications. The EB radiation effects on the ion transport properties of the polymer electrolytes due to substantial alteration of their chemical structure such as the displacement of atoms, creation of new double bonds, carbonization, chain scission (–C–C– bond scission) and radical–radical combination (crosslinking) were explained by Nouh *et al.* (2004) and Bauffard *et al.* (1995). These changes may promote the charge transport mechanism due to increased amorphous phases, wherein charges are easily trapped by adjacent ones. Further, this process is continuous and thus increases electrical conductivity as reported by Damle *et al.* (2008). EB irradiation also affects the optical properties of doped polymer electrolyte films.^5^ Polymers possess a high strength to weight ratio, easy processability and excellent physical properties, hence they are used in many applications like packaging, electric cable insulation, microelectronics, dosimeters and medical applications^6^ and also in the safety systems of nuclear power plants, insulation of superconducting magnets used in fusion reactors and in space applications after exposure to different ionizing radiation energies.^7,8^ There are a few results in the literature on EB irradiated polymers like polypropylene (PP), polyvinyl chloride (PVC), poly(vinylidene fluoride) (PVDF) that illustrate changes in thermal properties, structure, morphology and electrical conductivity after irradiation.^9,10^ The copolymer PVDF–HFP is considered a suitable host polymer due to its various interesting properties such as a high dielectric constant (*ε* = 8.4) which supports the dissociation of salt as low crystallinity can be helpful to improve ionic conductivity^11^ and it has excellent chemical stability due to the crystalline vinylidene (vdf) phase supported by a hexafluoropropylene (HFP) amorphous phase. The strong electron withdrawing functional group (–C–F) in PVDF–HFP makes this polymer anodically stable and modifications after its exposure to radiation have attracted more attention due to improved physical properties and the offer of new applications like opto-electronic devices, sensors, super capacitors, medical radiation therapy and food processing.^9,12^ The present paper reports the effect of EB irradiation on the optical and electrical properties of the copolymer PVDF–HFP doped with LiClO_4_ and an investigation into the change in chemical properties and morphology upon irradiation, confirming the decrease in optical band gap and increased ionic conductivity with increased EB dose. The polymer electrolytes exhibit the desired opto-electronic properties after exposure to radiation and hence irradiated PVDF–HFP/LiClO_4_ polymer electrolytes can be used in battery, solar cell and LED preparation.

## 2. Experimental methods

### 2.1. Materials

Poly(vinylidene fluoride-*co*-hexafluoropropylene) (PVDF–HFP, *M*_w_: 455 000 g mol^−1^) and lithium perchlorate (LiClO_4_, *M*_w_: 106.4 g mol^−1^) were purchased from Sigma Aldrich, USA and the dimethylformamide (DMF, *M*_w_: 73.09 g mol^−1^) used as a solvent was from Merck Millipore India.

### 2.2. Preparation of PVDF–HFP : LiClO_4_ polymer electrolyte films

The polymer electrolyte films were prepared using a solution casting method. PVDF–HFP : LiClO_4_ (90 : 10 w/w, PHL10) was dissolved in dimethylformamide (DMF–C_3_H_7_NO) solution separately and the solutions were stirred continuously for about 6–8 hours at room temperature. Then the mixture of both the solutions was further stirred for 12 hours to obtain a homogenous viscous solution. The solution was cast onto a Petri dish and allowed to evaporate at room temperature after which, a film was peeled off. The thickness of the free standing film is around 0.30–0.35 mm and was measured using a screw gauge.

### 2.3. Characterization techniques

Chemical changes were analysed using a Fourier transform infrared (FT-IR, model ALPHA BRUKER) spectrometer in the spectral range of 2000–500 cm^−1^ and a Bruker Ascend 400 MHz NMR spectrometer was used to record the ^1^H and ^13^C NMR spectra at room temperature using dimethyl sulfoxide (DMSO) as a solvent. The surface morphology was analysed using a Sigma Zeiss field emission scanning electron microscope (FESEM). The optical absorbance (*A*) was measured as function of wavelength (*λ*) in the range 190–1100 nm using a computerized double beam Perkin-Elmer Lambda-35 UV-visible spectrophotometer. The ionic conductivity measurements were done using a Wayne Kerr 6500B precision impedance analyser in the frequency range 40 Hz to 1 MHz at room temperature. The cyclic voltammetry analysis was done using a CHI-660E electrochemical workstation at room temperature.

### 2.4. Polymer electrolyte film exposed to 8 MeV energy electron beam irradiation

The PHL10 electrolyte films were irradiated using an 8 MeV energy electron beam (EB) with 260 mA of current at a 31 Hz pulse repetition rate as well as with a pulse width of 10 μS and a conveyor speed of 1.3 m min^−1^ scanning −4.0 A@200 ms at 40, 80, and 120 kGy dosage at room temperature at LINAC, Raja Ramanna Centre for Advanced Technology – Indore, India.

## 3. Results and discussion

### 3.1. FT-IR analysis

**Fig. 1:**
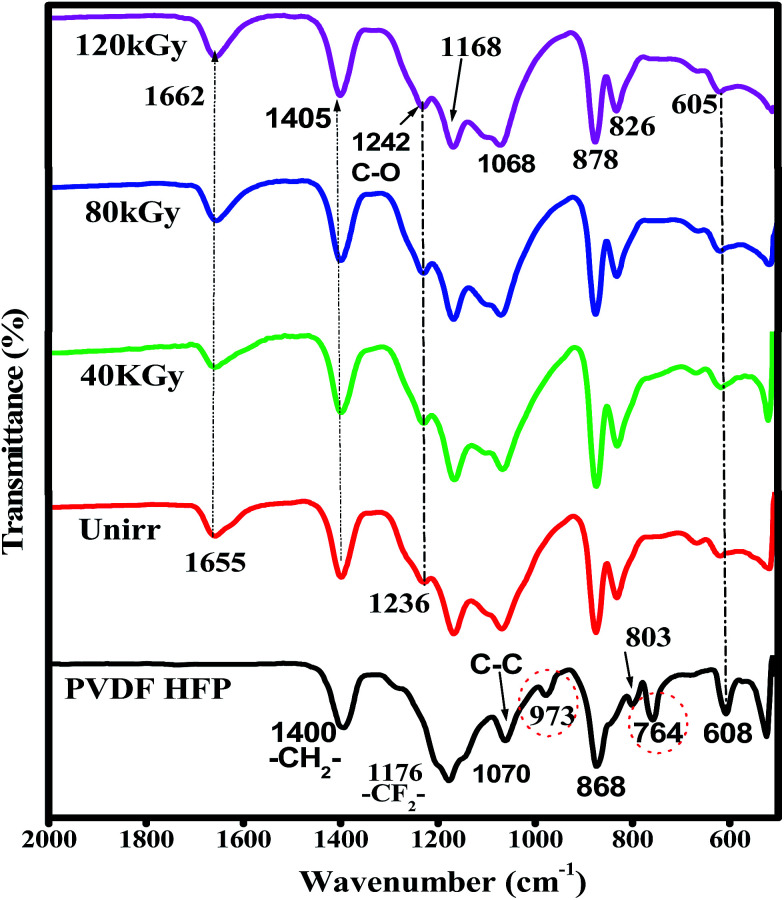
FT-IR spectra of pure PVDF–HFP, PHL10 electrolyte film before and after EB irradiation with different doses.

**Scheme 1:**
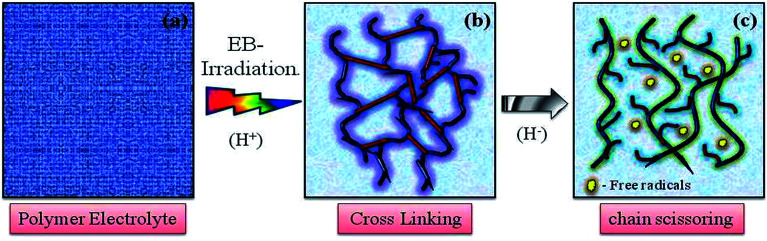
Representation of the cross-linking and chain scissioning mechanisms in polymer films.

**Scheme 2:**
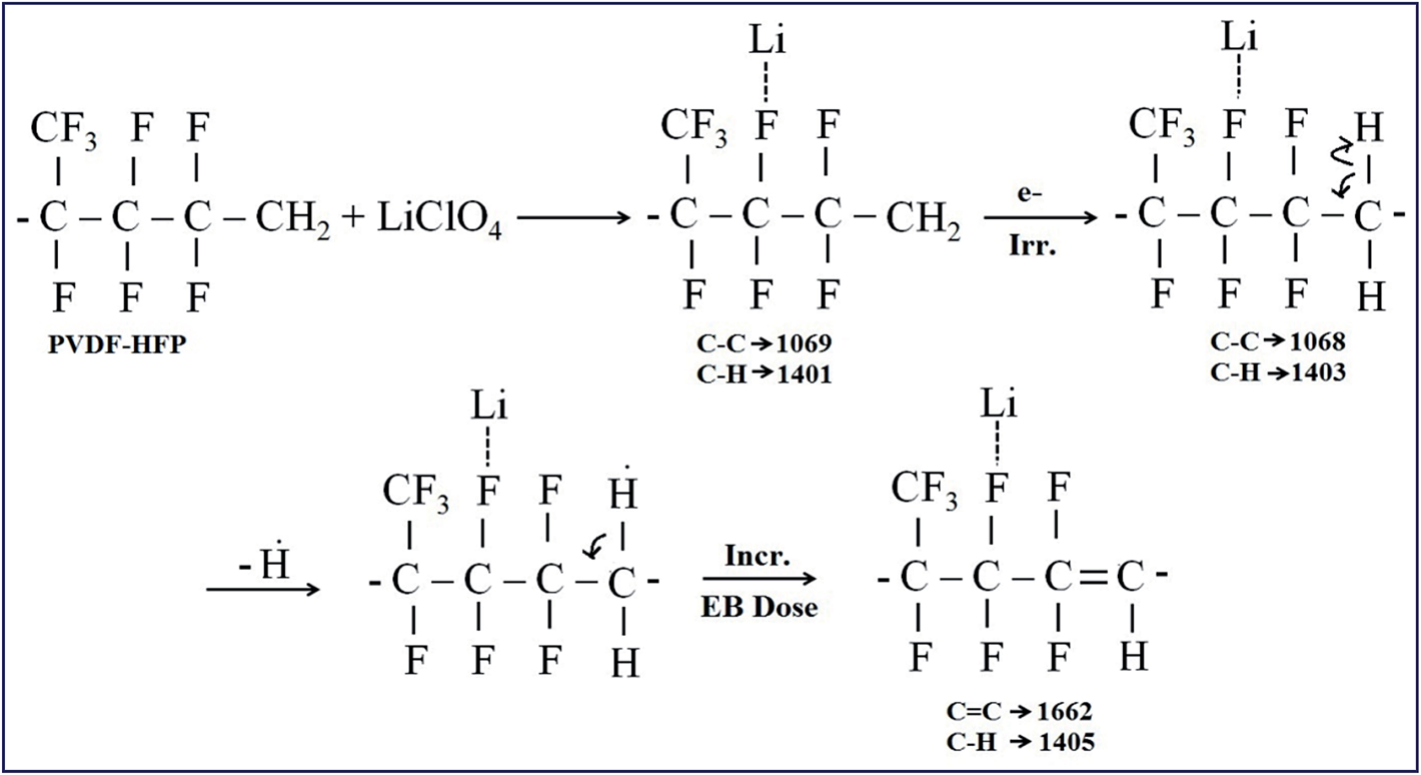
Representation of the formation of C=C bonds in polymer films.

The chemical changes due to EB irradiation were examined by analysing the changes in FT-IR peak intensity and shift compared with that of the unirradiated film, as shown in Fig. 1. From the spectra of PVDF–HFP, the crystalline phase VDF unit identified by the vibrational bands observed at 973, 803, 764 and 608 cm^−1^ has been shifted towards low frequencies at 870, 828, 763 and 610 cm^−1^ with reduced intensity, which confirms complexation between the salt and host polymer.^13,14^ Significant alterations in the FT-IR bands were observed after EB irradiation. The peak found at 1655 cm^−1^ in the unirradiated electrolyte, which has shifted to wavenumbers 1661, 1658 and 1654 cm^−1^ for 40, 80 and 120 kGy EB doses, respectively, is assigned to C=O bond stretching vibrations^15,16^ and confirms polymer chain degradation. The deformed CH_2_ band appearing at 1400 cm^−1^ is assigned to the vinylidene group of the host polymer and shifted to a higher wavenumber, 1405 cm^−1^, at 120 kGy due to C–H bond scissioning.^17,18^ It is clearly observed that the intensity of the CH_2_ bond wagging increases with radiation dosage due to dehydrogenation (breaking of H bonds) in the film, leading to C–H bond scissioning as a result of the formation of a C=C bond.^19^ The disappearance of the crystalline band at 978 cm^−1^ of PVDF–HFP after irradiation is clearly due to the deformation of the chemical structure and the amorphous peak at 868 cm^−1^ shifted to 878 cm^−1^, indicating increasing amorphous character due to the creation of defects in the polymer chain crystalline phase with higher doses. The chain scission phenomenon predominantly occurrs in the crystalline phase and reduction in the crystallite size upon EB irradiation has been reported.^17,20^Scheme 1 demonstrates the mechanism of cross-linking and chain scission due to EB irradiation. Scheme 2 shows the formation of C=C bonds in the polymer film after irradiation. Whenever the polymer electrolyte is exposed to a lower dosage, the breaking of bonds or C–C and C–H bond modification in the polymer was observed to occur through oxidative chain scission. As the EB dose increased, the C–H bonds undergo chemical deformation and form C=C bonds *via* a cross-linking process. The observed results confirm the effects of EB radiation on the polymer electrolyte.

### 3.2. Nuclear magnetic resonance (NMR) spectroscopy

**Fig. 2:**
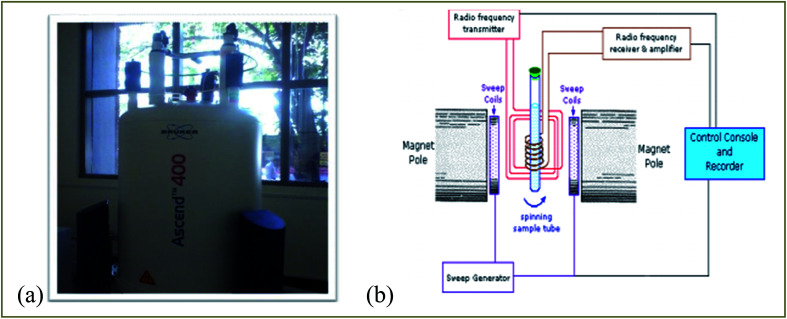
(a) Photograph of a Bruker Ascend 400 NMR spectrometer and (b) its block diagram.

**Fig. 3:**
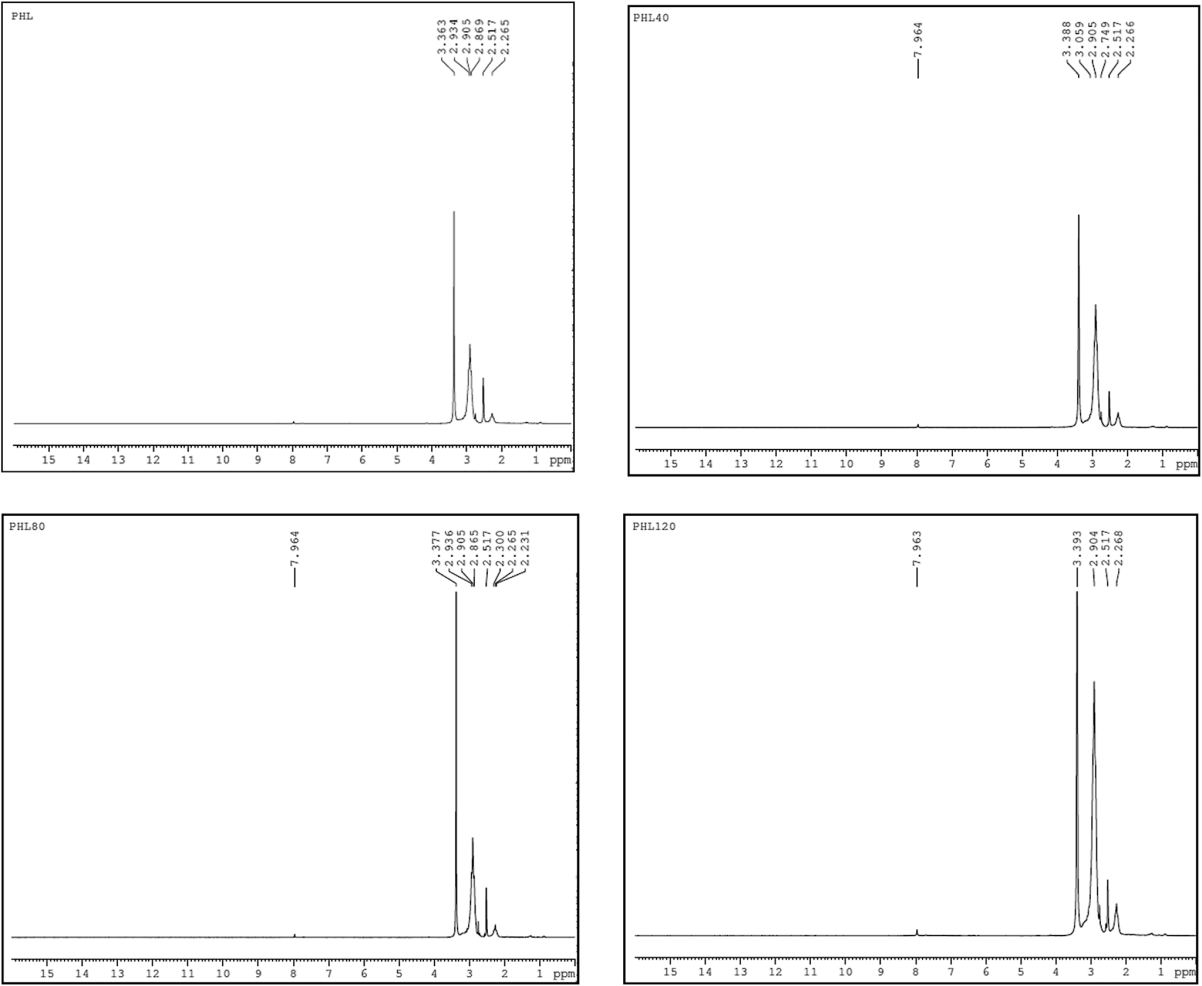
^1^H NMR spectra of unirradiated and EB irradiated PHL10 polymer electrolyte with different doses.

**Fig. 4:**
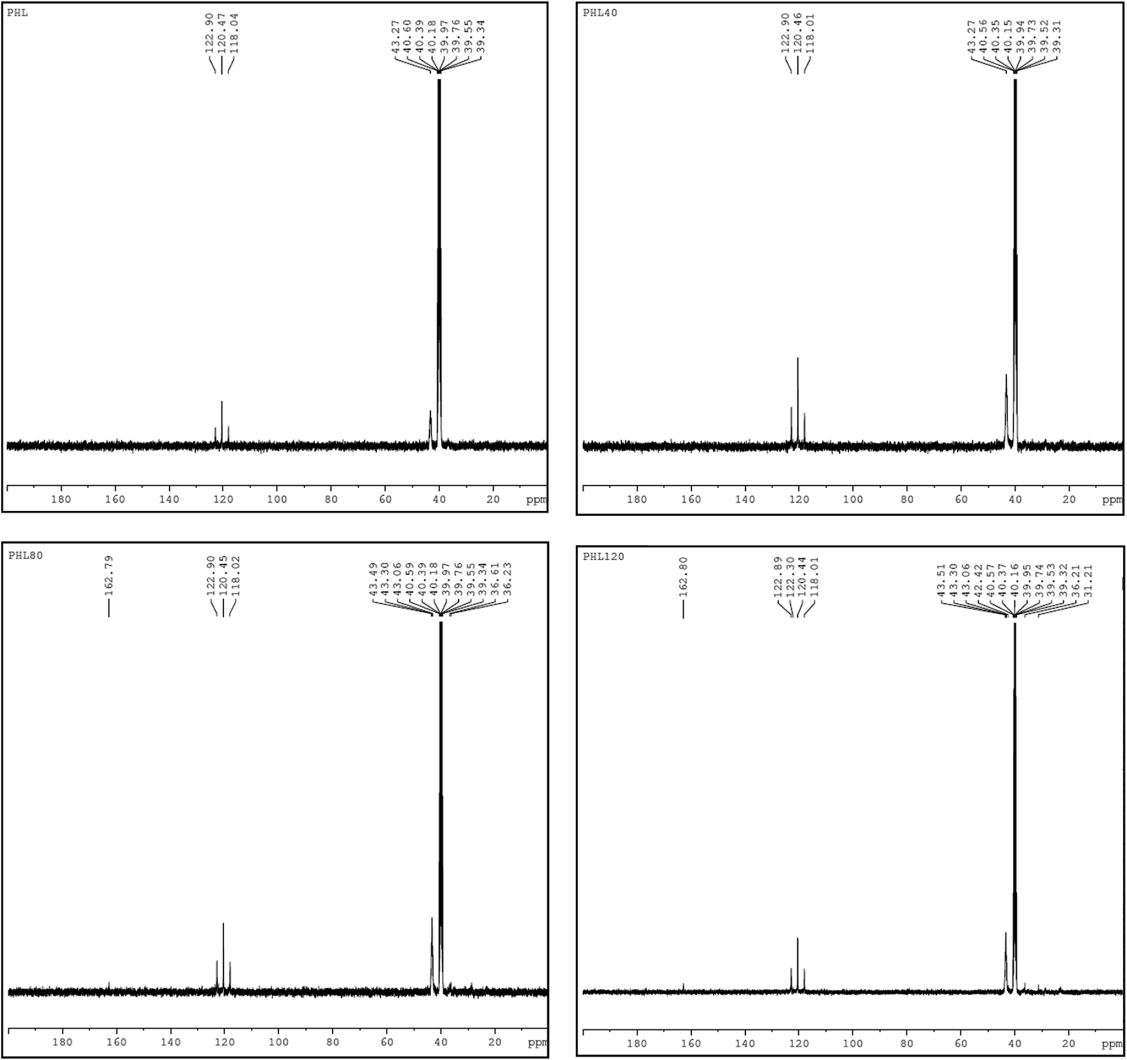
^13^C NMR spectra of unirradiated and irradiated PHL10 polymer electrolyte with different doses.

NMR spectroscopy is a well established powerful tool in the elucidation of polymer chain structure and dynamics. The typical ^1^H and ^13^C NMR spectra of unirradiated and EB irradiated PHL10 polymer electrolyte with 40, 80 and 120 kGy doses are presented in Fig. 3 and 4, respectively, and a photograph of the spectrometer and its block diagram are shown in Fig. 2.

There is a clear variation in the NMR spectra of the irradiated films and the steady increase is evident from the NMR shifts shown in Fig. 3 and 4. It was observed that there is a change in peak width, affecting not only the sharpness but also the splitting, with increasing EB dose. For unirradiated films, the peak is short. But, when exposed to 40, 80 and 120 kGy EB doses, the peak becomes very sharp and also split.^21^ The ^1^H NMR spectra of the PHL10 polymer electrolyte before and after EB irradiation reveal the absence of some spectral lines upon EB dosage, indicating the absence or loss of hydrogen through abstraction.^22^ Line narrowing is observed in the ^1^H NMR and ^13^C NMR spectra with increasing irradiation at room temperature due to the average intramolecular interactions. Further to this, the change is reflected in observed line narrowing due to the vanishing of intermolecular interactions, suggesting that there is ion mobility in the irradiated polymer electrolytes.^23^ The ^13^C NMR spectra confirm the effect of EB irradiation on the PHL10 polymer electrolyte through the sharpening and splitting of the spectral lines with increasing EB dose and also through the new spectral line at 162.80 ppm for the 120 kGy EB dose. The signal in the spectrum of the unirradiated film at around 43.27 ppm is shifted to 43.51 ppm after a 120 kGy EB dose and is assigned to carbon atoms. Other fluorinated carbon atoms in the PHL10 polymer electrolyte can be observed around the 118–122 ppm range. The carbon atoms in the CF_2_ groups of PVDF–HFP were assigned to the spectral lines between 118 and 121 ppm.^24^^13^C NMR offers good effective identification of resonance peaks assigned to various oxidation-induced functional groups affected by irradiation. The NMR study confirms the presence of the carbon groups and their relative chemical shifts and it has proven very useful in studying degradation and radiation-induced changes in polymeric materials.^25^ The results correlate with the FT-IR results.

### 3.3. Surface morphology of irradiated polymer electrolyte films

**Fig. 5:**
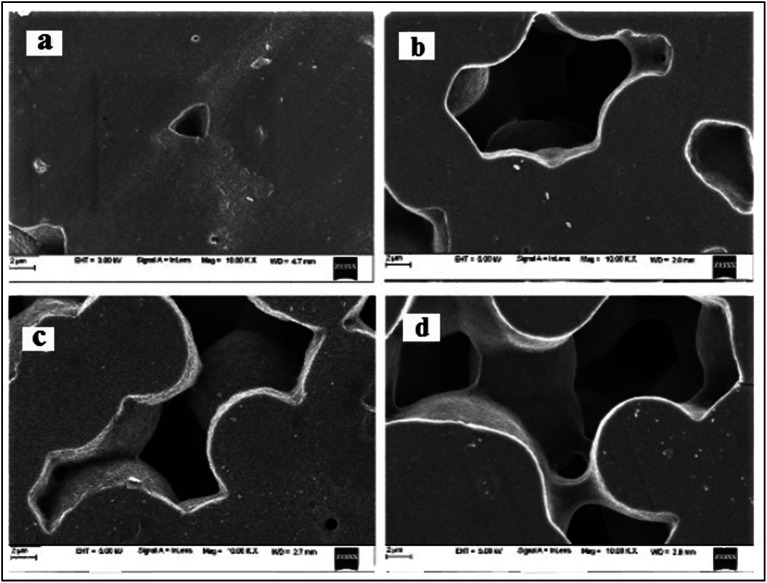
FESEM images of (a) unirradiated and EB irradiated with doses of (b) 40 kGy, (c) 80 kGy and (d) 120 kGy.

The FESEM images of PHL10 polymer electrolyte films before and after EB irradiation are shown in Fig. 5(a–d). A small triangle shaped porous structure was observed on the surface of the unirradiated film Fig. 5(a) and its size and shape drastically changed upon irradiation. The depth of the porosity increased after a 120 kGy EB dose, and the amorphous region increased as a result.^26^ In the case of the 120 kGy dose, it was also observed that the large porosity with an interior hollow inter-connecting network may provide conducting paths in which electrons could flow easily, thus increasing ionic conductivity.^27^ The change in morphology from rough to smooth and increasing pore size with increasing EB dosage suggests the degradation of the polymer matrix.^28,29^ The observed results confirm that the morphology can be altered by irradiation.

### 3.4. UV-visible absorption spectroscopy analysis

**Fig. 6:**
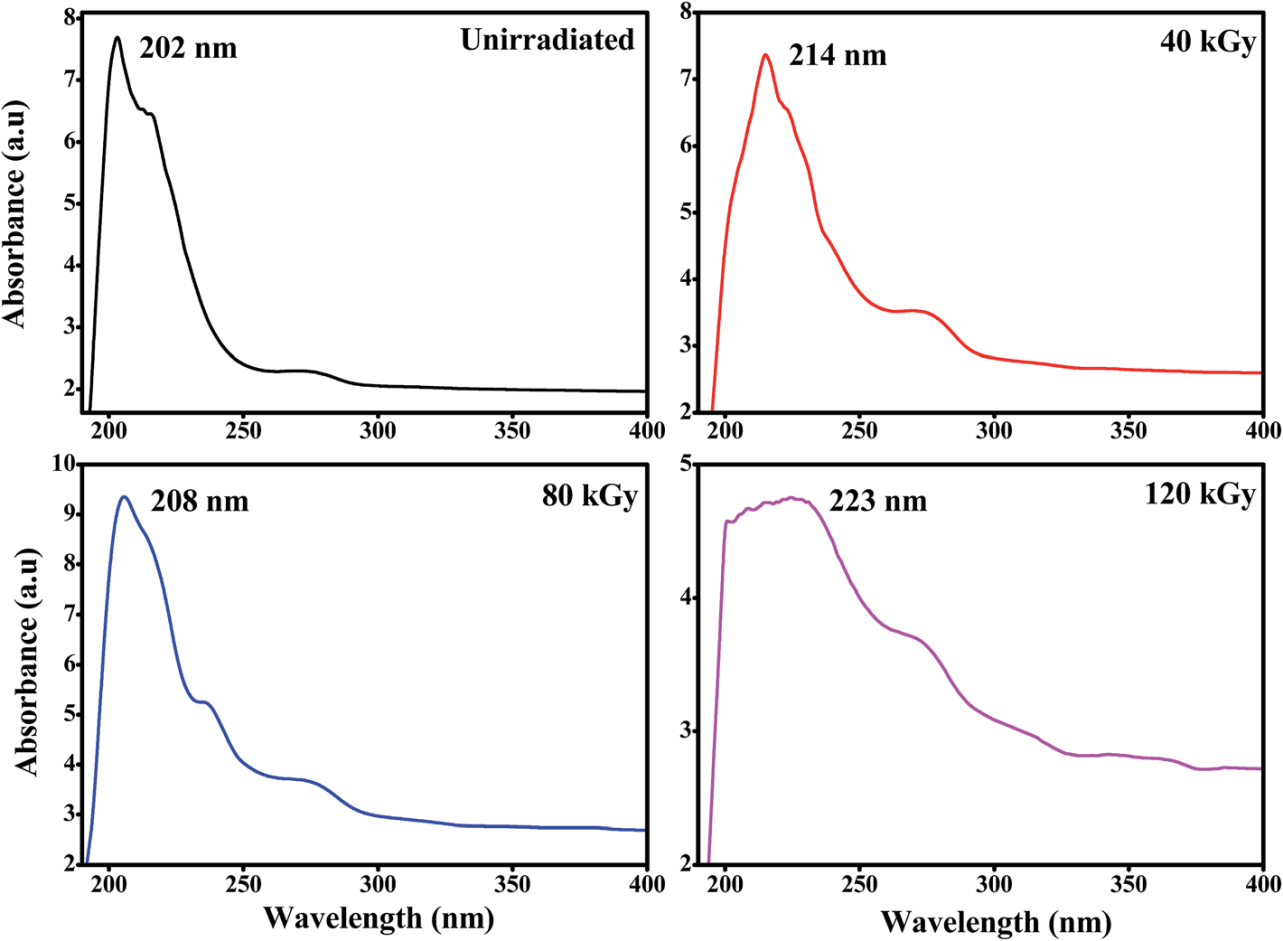
UV-visible spectra of the PVDF HFP/LiClO_4_ electrolyte before and after EB irradiation with 40 kGy, 80 kGy and 120 kGy doses.

**Fig. 7:**
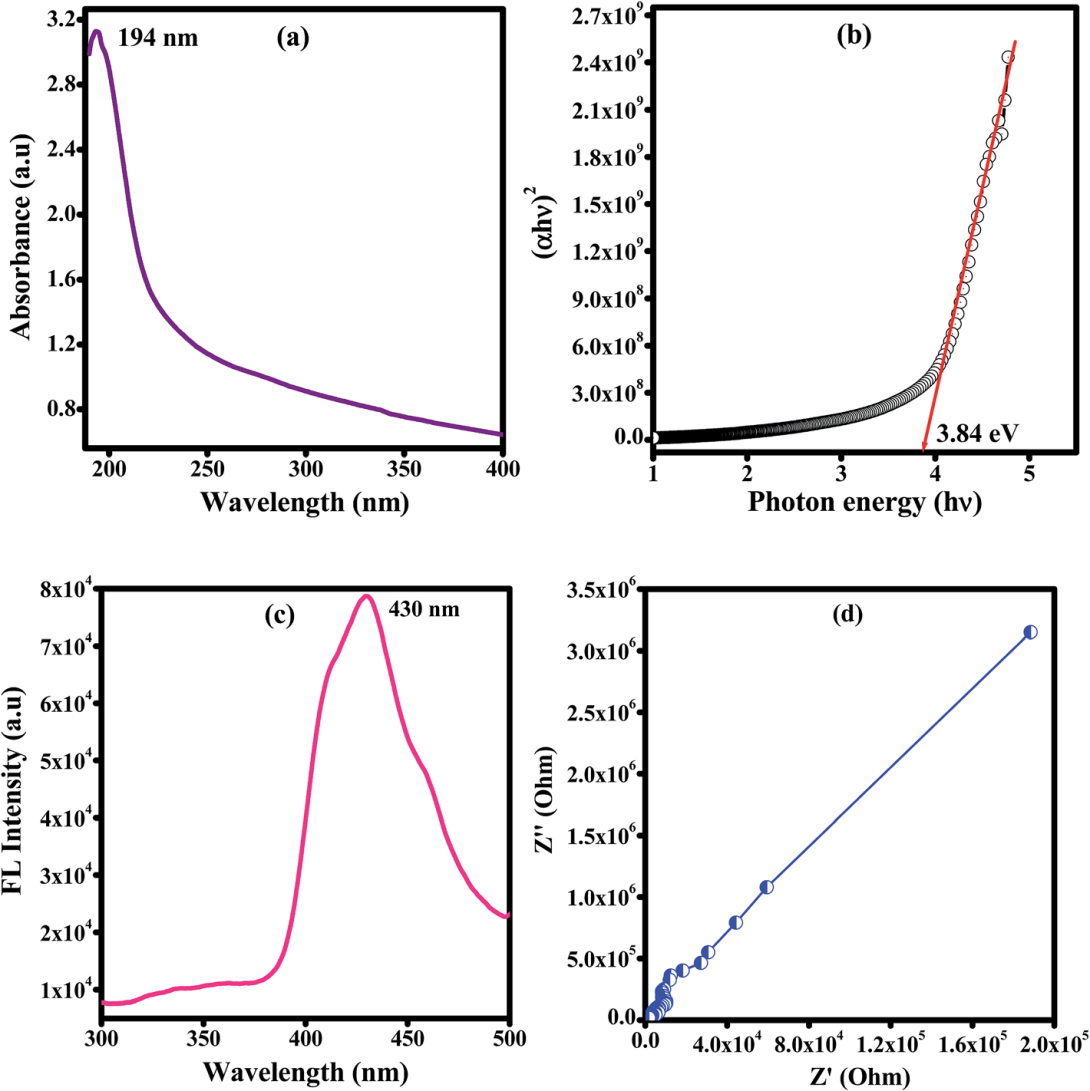
Plots for pure PVDF–HFP of the (a) UV-vis spectrum, (b) optical band gap, (c) fluorescence spectrum and (d) impedance spectrum measured at room temperature.

Fig. 6 shows the optical absorption spectra of unirradiated and EB irradiated PHL10 polymer electrolyte films. It can be seen that the absorption increases with increasing radiation dose which indicates an increasing number of dipoles.^19^ When EB radiation energy interacts with a polymer electrolyte, it may undergo radiation processes to produce fragmentation (defects) in the polymer chain.^19,30,31^ The absorption peak of PVDF–HFP found at 194 nm (Fig. 7(a)) is shifted to 202 nm for the unirradiated PHL10 film and similar results have been reported previously.^32^ The absorption spectra of 40, 80 and 120 kGy dose irradiated films show two absorption bands in the range 214–223 nm and 269–274 nm as shown in Fig. 6.

The absorption wavelength has been shifted to 214, 208 and 223 nm for 40, 80 and 120 kGy doses, respectively, in the UV region, which indicates a decrease in forbidden band width due to the alteration of C=O groups in the irradiated polymer electrolyte.^19,33^ The absorption bands in the second stage are shifted to 265, 271 and 274 nm with increasing EB doses of 40, 80 and 120 kGy, respectively, and clearly indicate that there are increased inter–intra band transitions of electrons, forming sub-energy levels within the existing band gaps.^34,35^ It could also be interpreted that the single C–C bond may convert into a C=C bond due to hydrophobic processes (H-bond breaking) with a high dose, confirming the degradation of the polymer.^36^ The presence of double bonds along the polymer chain can decrease the binding energy of the polymer and produce radiation induced conjugated C=C bonds continuously and as a result, single bonds (194 nm) disappear to produce double (223 nm) and triple (274 nm) bonds^34,37^ which may be attributed to polymer chain scissioning^38^ resulting from a decrease in the band gap energy after irradiation.

### 3.5. Optical band gap energy of PHL10 polymer electrolyte

**Fig. 8:**
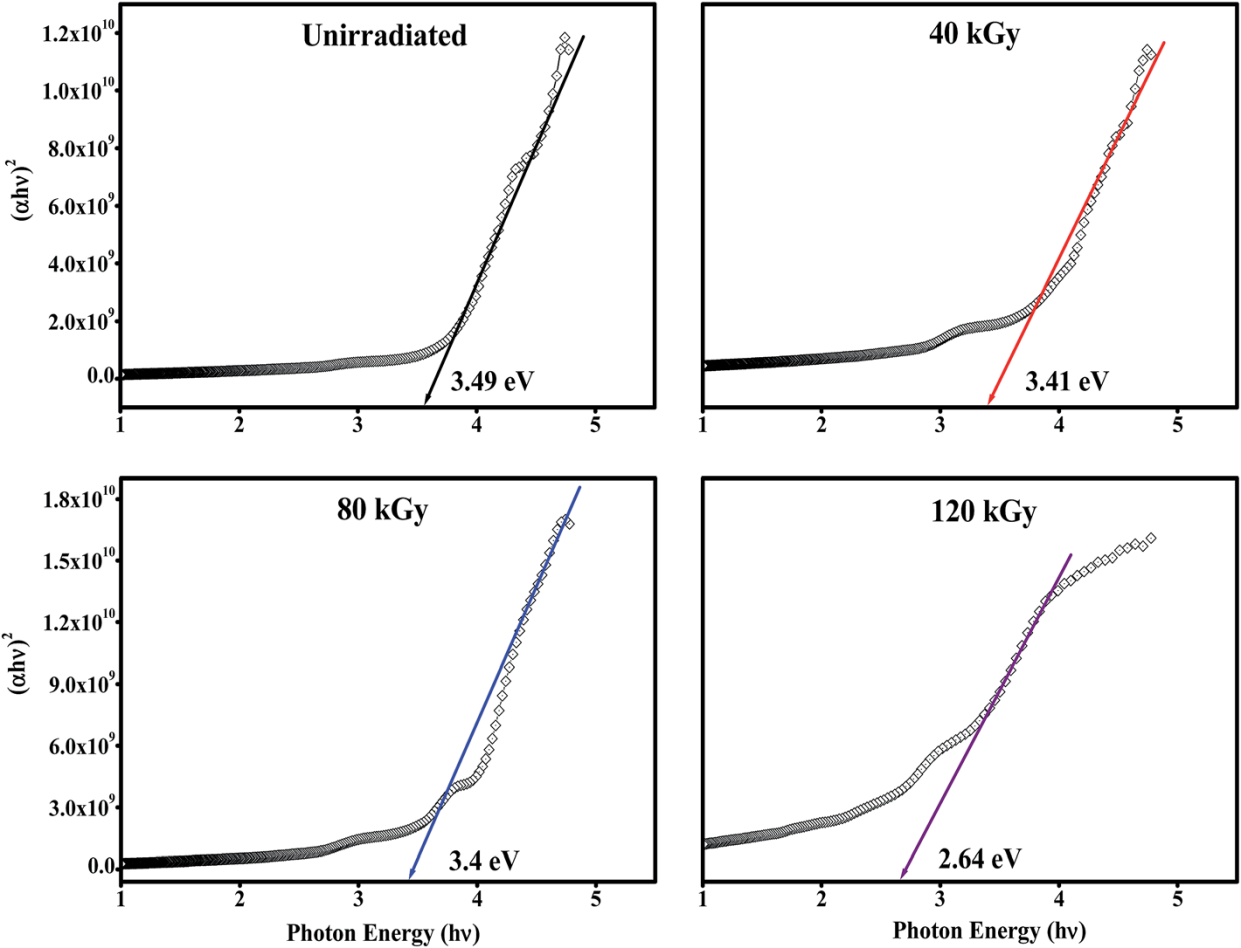
(*αhν*)^2^*versus* photon energy (*hν*) plots of PHL10 electrolyte films before and after EB irradiation with different dosage.

The optical band gaps of EB irradiated and unirradiated PHL10 electrolyte films were calculated using the relation between the absorption coefficient (*α*) and incident photon energy (*hν*) given by the following equation,1
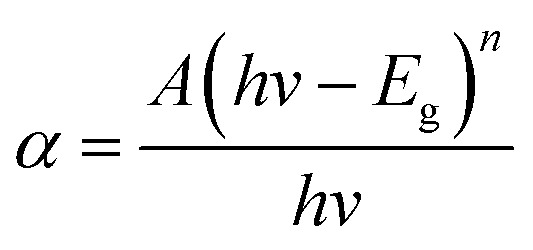
where, *E*_g_ is the energy bad gap in eV, *A* is a constant, *n* is the exponent and 1/2 for directly allowed transitions. The absorption of light *I* = *I*_0_ exp(−*αX*) was used to calculate the absorption coefficient according to the Davis and Mott (1979) model, where *α*(*λ*) is the absorption coefficient which can be calculated from the optical absorption spectrum using the relation,2
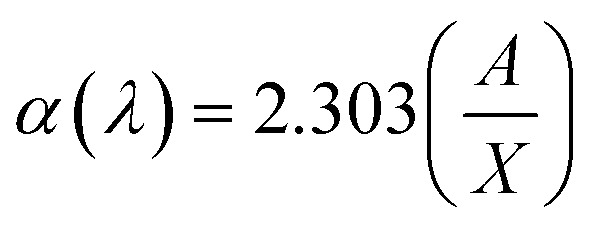
where, *λ* is the wavelength of the incident photons, *A* is defined as *A* = log(*I*_0_/*I*), where *I*_0_ and *I* are the intensities of the incident and transmitted beams respectively and *X* is the thickness of the sample.^39^ The optical band gap values are extracted from the linear portion of the (*αhν*)^2^*versus hν* plots shown in Fig. 8. The optical band gap of the host polymer is 3.84 eV (Fig. 7(b)) and was found to decrease to 3.49 eV in PHL10 film, which was attributed to the delocalization of Li^+^ ions in the polymer matrix^40^ as shown in Fig. 7(b).

The optical band gap shows a decreasing trend, with values of 3.41, 3.4 and 2.64 eV at 40, 80, and 120 kGy dosages, respectively. This is due to the formation of defects, fragmentation, free radicals and the creation of traps in the polymer electrolyte at higher dosages.^34^ The decrease in optical band gap upon EB dosing confirms the creation of more free electrons in the conduction band, resulting in high conductivity. These results are in good correlation with the morphological results.

### 3.6. Fluorescence spectroscopy analysis

**Fig. 9:**
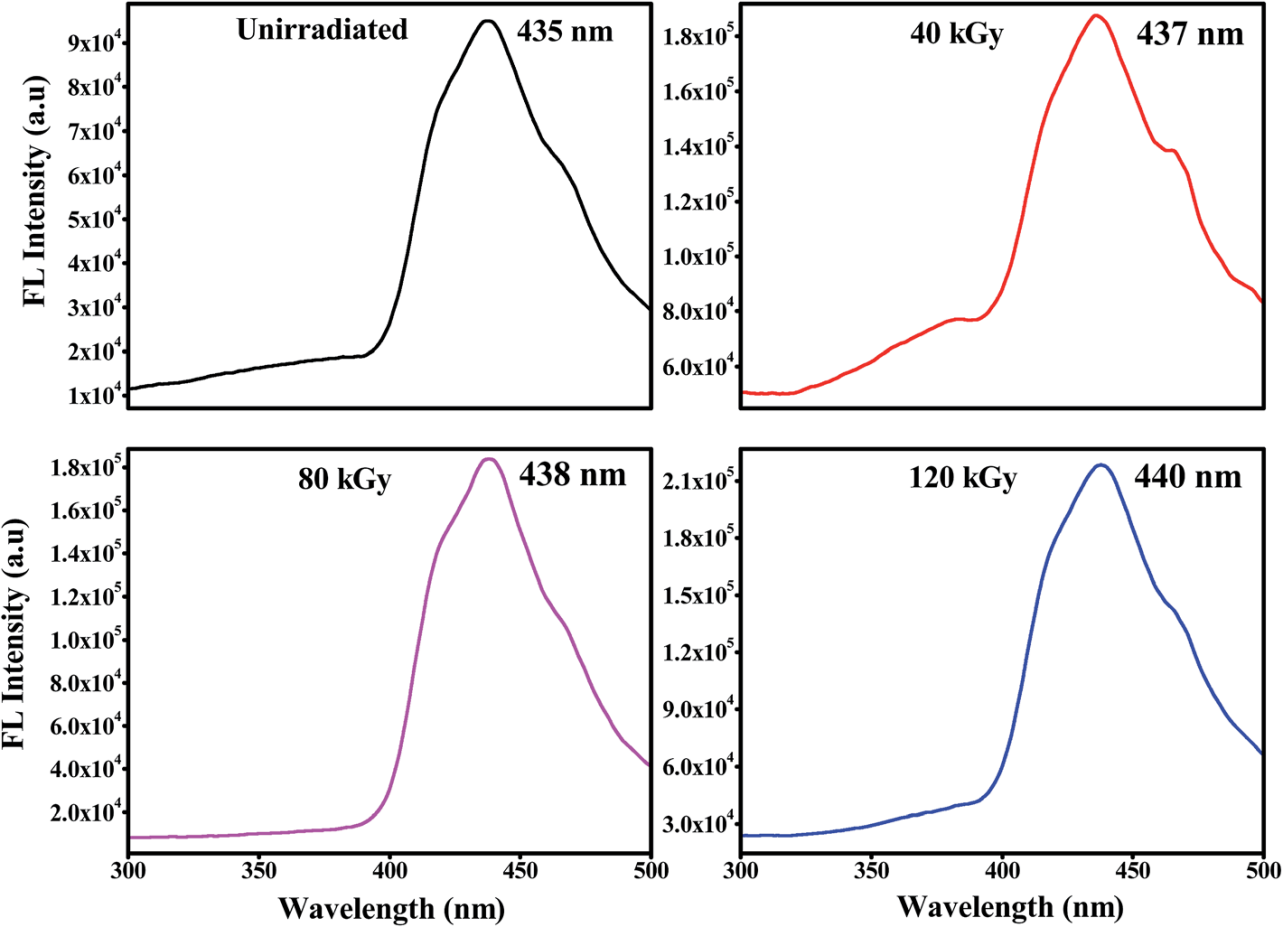
Fluorescence excitation spectra of PHL10 electrolyte films before and after EB irradiation at an excitation wavelength of 210 nm.

The fluorescence emission spectra of PHL10 electrolyte films before and after EB irradiation at different dosages are given in Fig. 9 and that of pure PVDF–HFP in Fig. 7(c). The fluorescence spectra demonstrate the energy excitation and emissive properties of the materials. It can also indicate that ion mobility in the polymer structure is related to charge carriers surrounded by local polarity in the porous structure of the polymeric media.

From Fig. 9 it is clear that, as EB dose increases, there is an increase in intensity for all doses, suggesting an enhancement in the molecular motion or segmental motion in the polymer system as a result of increasing ionic conductivity.^41,42^ The PVDF–HFP peak positioned at 430 nm (Fig. 7(c)) shifted to 435 nm for unirradiated polymer electrolyte films as shown in Fig. 9(c). As EB dose increases, the fluorescence spectra exhibit red-shifting and broadening of the vibronic bands. The peak wavelength shifted from 435 nm for unirradiated films to 437, 438, and 440 nm for 40 kGy, 80 kGy and 120 kGy doses, respectively. This red-edge effect was attributed to defect states in the polymer matrix, indicating a decrease in the energy gap between the highest occupied molecular orbital (HOMO) and the lowest unoccupied molecular orbital (LUMO). This is also consistent with the decrease in the optical band gap energy with increasing dose, leading to an increase in the mobility of the ions, which translates into increased conductivity.^43–45^ The observed results are strongly consistent with the red-shifts observed in the absorption spectra shown in Fig. 6.

### 3.7. Ionic conductivity study

**Fig. 10:**
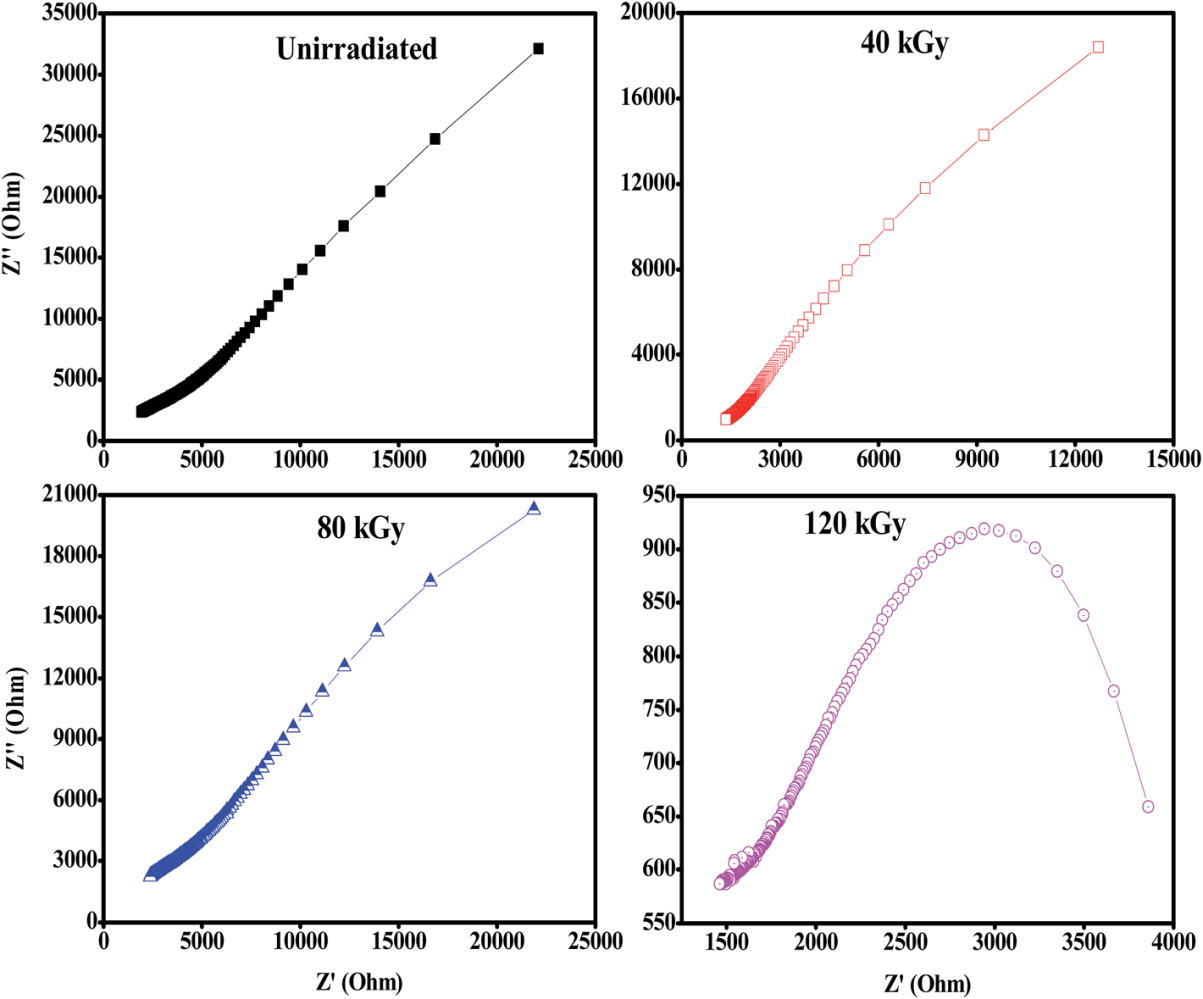
Nyquist plots of PVDF HFP/LiClO_4_ electrolyte before and after EB irradiation measured at room temperature.

The ionic conductivity of the polymer electrolyte films was measured using an impedance analyser using a two probe method in the frequency range of 40 Hz to 1 MHz. Fig. 10 shows the complex impedance plots of the PHL10 electrolyte films before and after EB irradiation at different dosages at room temperature. For a symmetric cell, there should be two types of semicircles in the impedance spectrum: the bulk electrolyte impedance corresponding to high frequencies, and the interfacial impedance related to low frequencies, according to Watanabe and Ogata.^46^ In the literature, it has been reported that at high frequency, if the semicircle does not appear for the polymer electrolyte, it indicates that the conductivity is mainly due to the ions.^47^ It is seen that initially there is no semicircle in the impedance plots for PHL10 electrolyte films, but the exhibited straight line in the lower frequency region is associated with Li^+^ diffusion. But, as the irradiation dose increased to 120 kGy it shows a clear semicircle at the high frequency, indicating enhanced ion mobility in the polymer electrolytes. This is a result of the segmental motion permitting the ions to hop from one site to another site due to the contact resistance, including Li-ion migration, attributed to the charge-transfer reaction in the electrolyte interface with the same phenomenon reported for other polymer systems.^48^

**Fig. 11:**
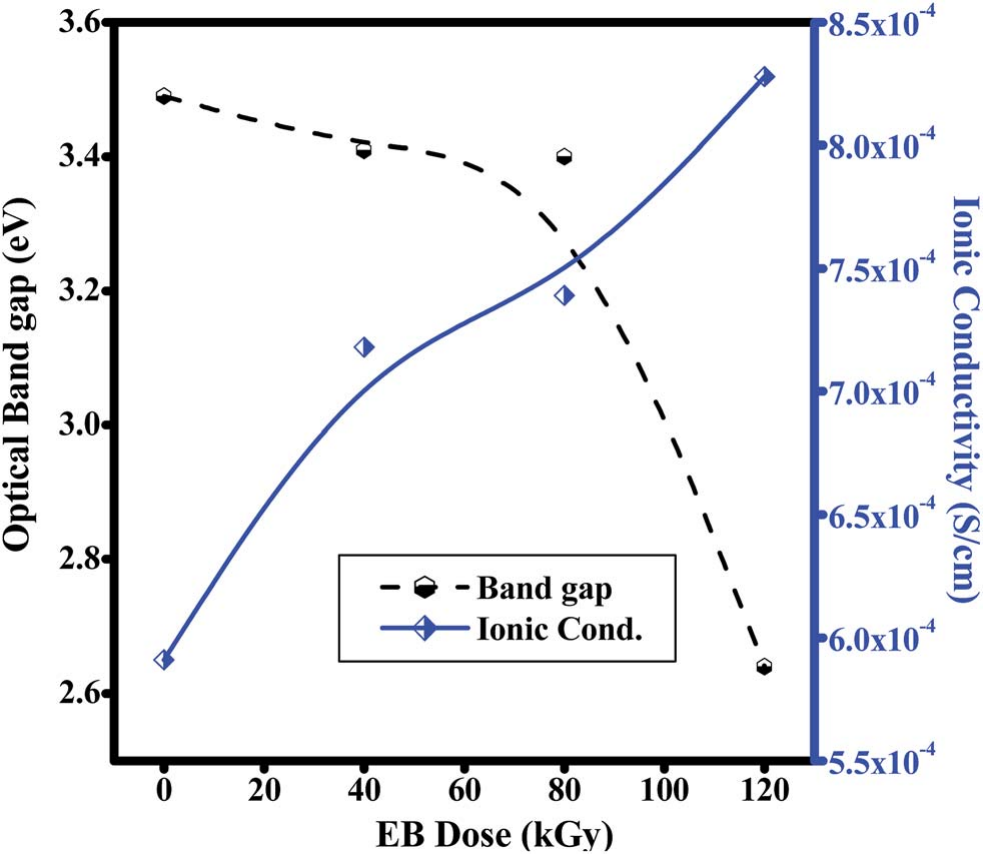
Plots of ionic conductivity and optical band gaps of PHL10 electrolyte films against EB dosage.

Fig. 11 shows increasing ionic conductivity and decreasing optical band gap with dose.^49^ The ionic conductivity in S cm^−1^ of each electrolyte was calculated using eqn (3),3
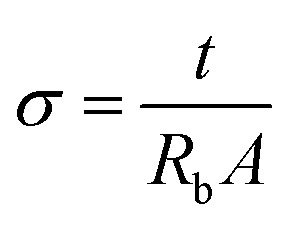
where, *t* is the thickness of the electrolyte film, *R*_b_ is the bulk resistance and *A* is the area of the electrolyte–electrode contact. The imaginary impedance (*Z*′′) was plotted against the real impedance (*Z*′) and the bulk resistance (*R*_b_) was obtained from the intercept with the real axis at higher frequency. The calculated ionic conductivity values are listed in Table 1 and the highest ionic conductivity of about 8.28 × 10^−4^ S cm^−1^ at room temperature was achieved with a 120 kGy dose, which is higher than that of other reported values at room temperature.^50^

**Table 1:** Ionic conductivity (*σ*) and optical band gap energy for PVDF HFP before and after EB irradiation

Sample name	Ionic Cond. (×10^−4^, S cm^−1^)	Optical band gap (eV)
PVDF–HFP	1.81	3.84
PHL Unirr.	5.91	3.49
PHL 40 kGy	7.18	3.41
PHL 80 kGy	7.39	3.40
PHL 120 kGy	8.28	2.64

### 3.8. Cyclic voltammetry studies

Cyclic voltammetry (CV) was performed to investigate the effect of EB irradiation on the electrochemical performance of PHL10 polymer electrolytes before and after EB irradiation using a CHI-660E instrument with a three electrode system. Pt wire was used as a counter electrode, Ag/AgCl as a reference electrode and glassy carbon as a working electrode placed in a solution of the film dissolved in 10 ml of DMF solvent.

The typical CV characteristic curve of the PHL10 polymer electrolyte with different EB doses was measured at a scan rate of 200 mV s^−1^ and with a −3 to 1 V potential window. The electrochemical performance increased with increasing radiation dose and reached a maximum with a 120 kGy dose, confirmed by the increased redox reaction at the cathodic peak around −0.9 V due to the uniform distribution of Li^+^ ions. The high radiation may prevent the aggregation of ions in irradiated electrolytes resulting in increased active surface area under the CV curves. Hence, the electrochemical stability of the polymer electrolytes can be improved with increasing EB dose.^51,52^

**Fig. 12:**
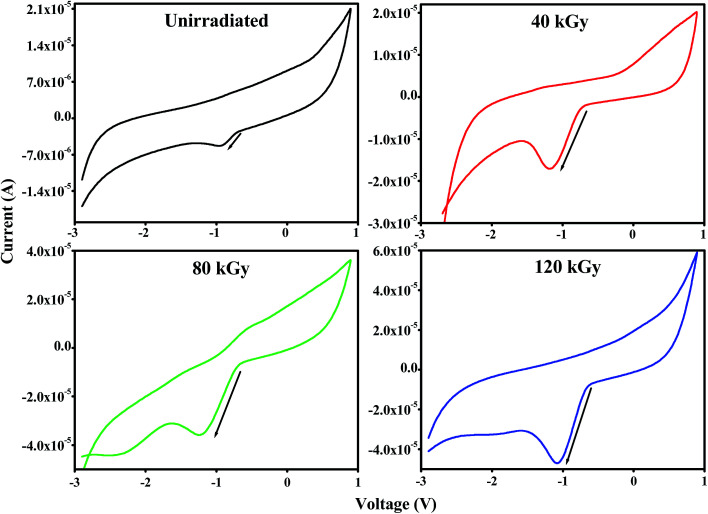
Cyclic voltammetry (CV) curves of PVDF HFP/LiClO_4_ polymer electrolytes before and after EB irradiation.

From Fig. 12, it is clear that the increased peak currents and potential peak separation (redox peak) for the increased EB dose suggests a fast electron transfer at the interface of the irradiated electrolyte^53,54^ which is very good for potential applications, especially in Li-ion rechargeable battery applications.

**Fig. 13:**
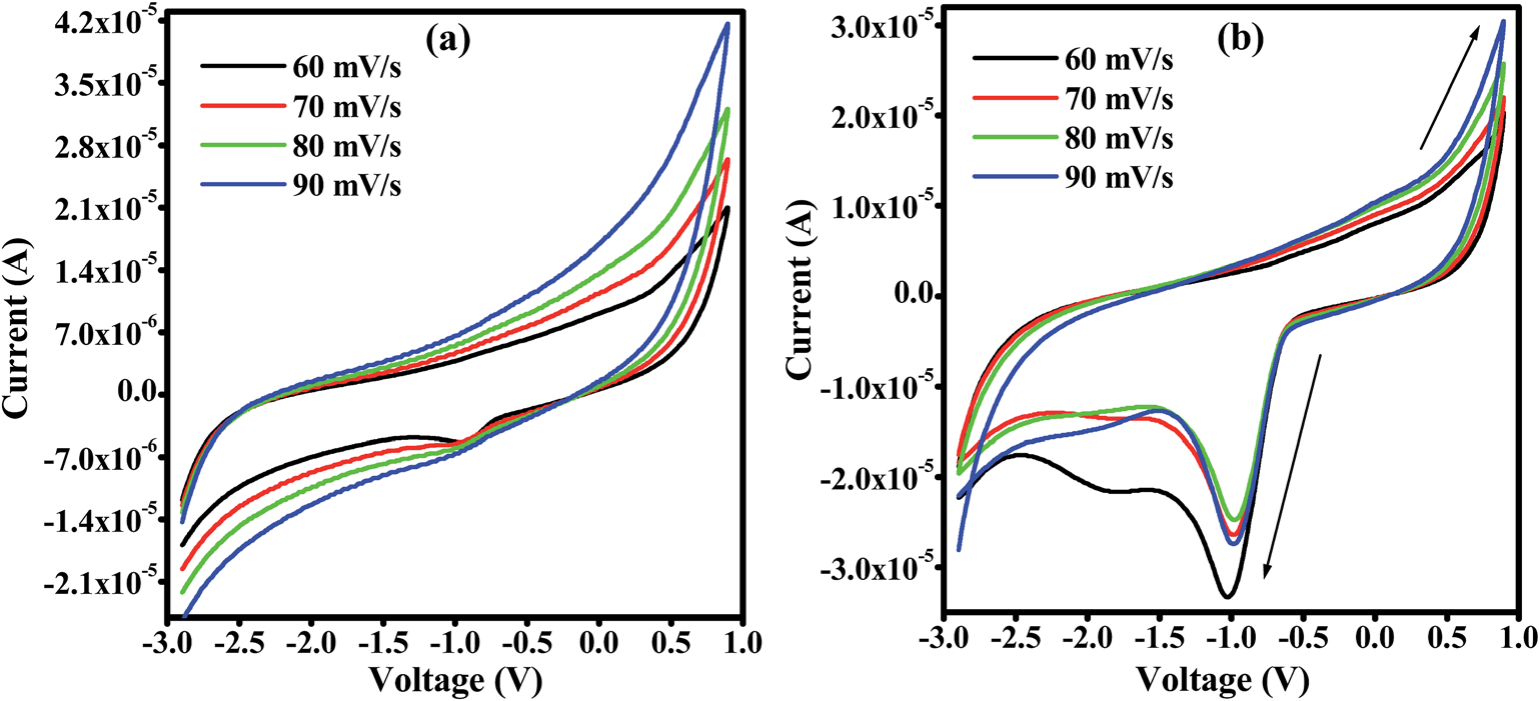
Cyclic voltammetry (CV) curves of PVDF HFP/LiClO_4_ polymer electrolyte films at different scan rates (a) unirradiated (b) 120 kGy EB dose.

CV curves for unirradiated and 120 kGy EB irradiated PHL10 polymer electrolytes were obtained between potentials of −3 and 1 V at different scan rates of 60, 70, 80 and 90 mV s^−1^ at room temperature and are shown in Fig. 13(a and b). The CV results indicate the capacitance nature of polymer electrolytes at high scan rates. The unirradiated polymer electrolyte shows an ideal shape at all scan rates, Fig. 13(a); there is no visible peak over the potential region. This indicates that charge and discharge occur reversibly at the electrolyte interface.^55^ When the polymer electrolyte is exposed to a 120 kGy EB dose Fig. 13(b), the CV curve exhibits reversible humps at all scan rates, revealing the redox reaction and confirming the capacitive behavior.^56^ Also, with increasing scan rate, the oxidation and reduction peak currents increase significantly.^57^ The observed results suggest that the polymer electrolytes are electrochemically active and an active electrolyte for lithium ion battery applications.

## 4. Conclusions

The prepared PHL10 electrolyte films were exposed to EB radiation at different dosages. The FT-IR results confirmed complex formation and irradiation effects through shifting peak positions and varying peak intensity due to chain scissioning or cross-linking processes. The NMR study confirms the relative chemical shifts and degradation and radiation-induced changes in the polymeric materials. The surface morphology was transformed into one with deep and large pores after irradiation, indicating the degradation of the polymer matrix upon irradiation. The absorption bands are red shifted with increasing EB dose, clearly resulting from increasing inter–intra band transitions of electrons forming sub-energy levels within the existing band gaps. For this reason, the optical band gap of 3.49 eV for unirradiated films decreases to 2.64 eV after exposure to a 120 kGy EB dose. The fluorescence results show a shift in wavelength from 430 nm to 440 nm with increasing intensity, indicating increased segmental motion of the polymer chains after irradiation. The bulk impedance plots for polymer electrolyte films reveal a semicircle at high frequency indicating Li-ion migration through the porous surface in the irradiated electrolyte films. CV curves reveal the redox reaction at different scan rates and confirm the capacitive behaviour and also that PHL10 polymer electrolytes are electrochemically active materials for lithium ion battery applications after irradiation. These observations will conclude that the physical properties of the polymer electrolytes can be tuned by electron beam irradiation at different doses for commercial applications, such as opto-electronic applications like LEDs, solar cells *etc.*

## Conflicts of interest

There is no conflicts of interest.

## Supplementary Material
